# Effects of a training program on person-centered care in nursing homes rated by residents: a quasi-experimental design

**DOI:** 10.1186/s12877-025-06340-7

**Published:** 2025-10-23

**Authors:** Lijuan Xu, Annakarin Olsson, Kewen Zhu, Zanhua Zhou, Maria Engström

**Affiliations:** 1 Medicine College, Lishui University, No.1 Xueyuan Road, Lishui City, China; 2 Department of Caring Science, Faculty of Health and Occupational Studies, University of Gävle, Kungsbäcksvägen 47, Gävle, 801 76 Sweden

**Keywords:** Nursing, Nursing homes, Person-centered care, Residents, Staff, Training program

## Abstract

**Background:**

The outcomes of staff training programs regarding the effect of person-centered care in nursing homes as rated by their residents are limited. This study examines the effects of a nursing home training program by evaluating resident-rated person-centered care, comparing changes over time between an intervention group and a comparison group.

**Methods:**

This quasi-experimental study was conducted in 25 nursing homes. A total of 124 residents in the intervention group and 65 in the comparison group were enrolled between August 2021 and June 2023. Data were collected using the ‘Person-centered Climate-Questionnaire-Patient version’ (twice, pre- and post-intervention). The two-year training program comprised knowledge and skills in quality of care through lectures, seminars, and skill practice among 15 managers and frontline staff members in nursing homes. These staff members were expected to implement what they had learned and diffuse their knowledge to their coworkers at their nursing homes. An ANCOVA analysis was performed with a significance level of *p* ≤ 0.05.

**Results:**

Among the residents, the person-centered scores were statistically significant (*p* < 0.001). The intervention group scores improved over time (mean = 13.6, SD = 20.0), whereas the comparison group declined (mean=-5.9, SD = 24.6).

**Conclusion:**

The program was associated with improvements in person-centered climate as rated by residents. These findings highlight the clinical importance of training programs, including knowledge and skills in person-centered care, dementia care, teamwork, staff empowerment, and psychological nursing care, is central for improving person-centered care for nursing home residents.

**Trial registration:**

Chictr.org.cnChiCTR2100048628. Registered July 12, 2021.

**Supplementary Information:**

The online version contains supplementary material available at 10.1186/s12877-025-06340-7.

## Introduction

Person-centered care (PCC) has gained considerable attention as a paradigm for improving the quality of life of older people living in nursing homes. This approach emphasizes individual needs, preferences, and values, as opposed to the traditional models of care that are often task-oriented and standardized [1–3].

PCC is based on the Eight Picker Principles of Person-Centered Care [4], including “*fast access to reliable healthcare advice; effective treatment by trusted professionals; continuity of care and smooth transitions; involvement and support for family and carers; clear information and support for self-care; involvement in decisions and respect for preferences; emotional support*,* empathy and respect; and attention to physical and environmental needs*”. These principles align with the broader goals of geriatric care with dignity, autonomy, and holistic well-being at the focal point. PCC aims to foster a supportive environment in which older people feel respected, valued, and involved in their own care decisions [5]. An increasing number of studies have been conducted on PCC interventions for older people in nursing homes, focusing on environmental enhancement, improved interaction skills, management and leadership skills, nurse empowerment, and individualized care cultures [6, 7]. The results indicate positive effects, such as improved quality of life and mental health among residents, as well as staff job satisfaction and PCC competence [8–10]. In a review by Pakkonen et al. [7] most PCC interventions in nursing homes were effective when evaluated by staff or proxies. However, studies that place resident ratings as pivotal are sparse.

The use of PCC in nursing homes is challenging because of structural, cultural, and resource-related barriers [11–13]. The most important obstacles reported are organizations without implementation plans, few training opportunities, and an unstable context for PCC [11].

Previous studies have highlighted the potential benefits of PCC training programs and/or value-based training programs in nursing homes, with improved resident satisfaction [10, 14], a person-centered climate [10, 15], quality of daily activities [15], enhanced quality of care [14], an increase in staff job satisfaction [7] and PCC competence [9]. However, studies that consider residents’ perspectives and structured implementation strategies at the organizational level are quite few. There has been a reliance on proxy rating for residents; however, more studies are needed wherein residents in nursing homes evaluate the effectiveness of a training program. Therefore, this study aimed to evaluate the effect of a nursing home staff training program—developed following the Implementation Methods of Training for Senior Service Personnel (Lishui Civil Affairs Bureau), on resident-rated person-centered care (PCC), comparing changes over time between the intervention and comparison groups.

## Materials and methods

### Design

The study design was quasi-experimental, with a non-equivalent control group, and a pretest-posttest design for the residents.

### Samples and settings

**Fig. 1:**
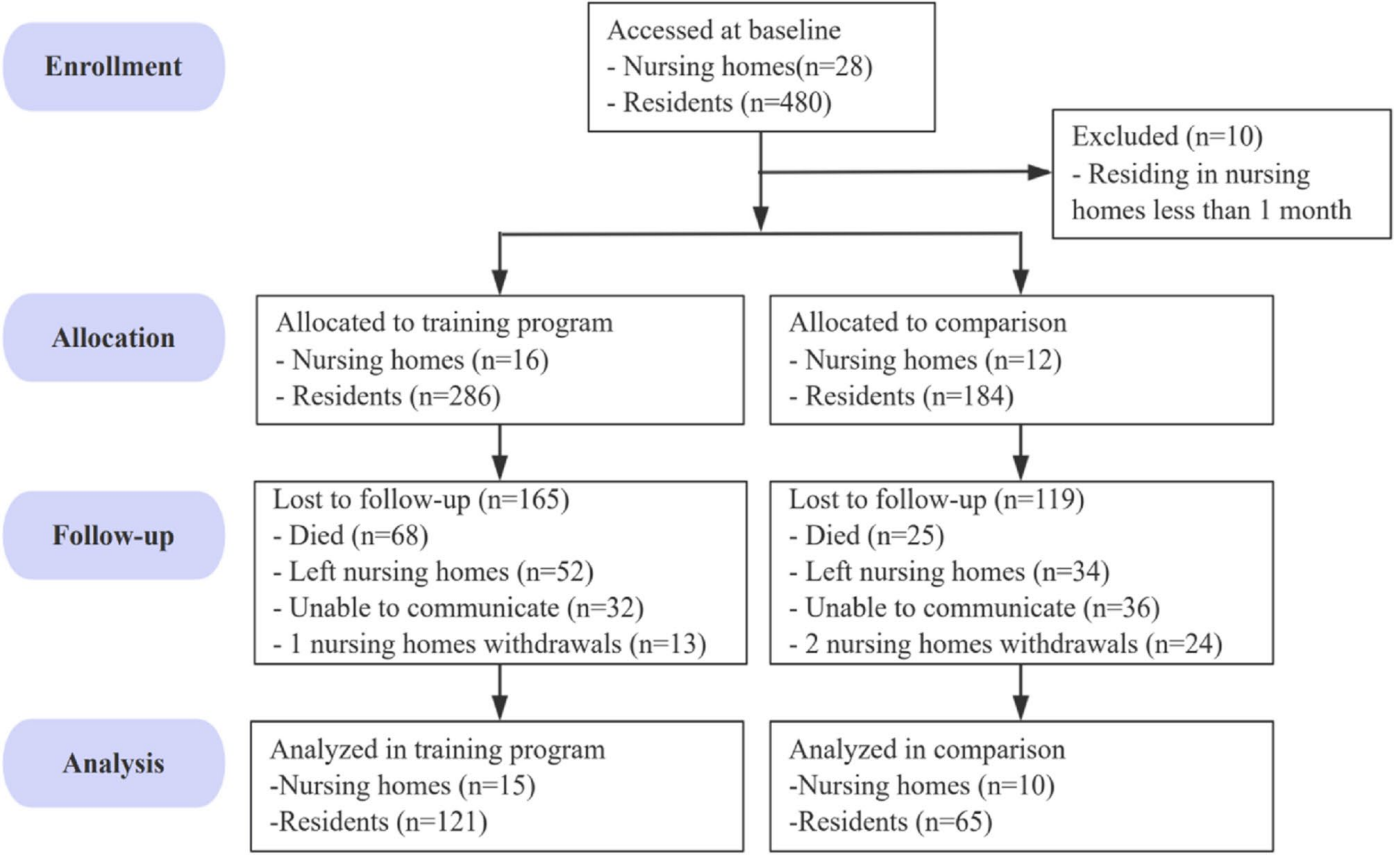
Flowchart of the study participants

At baseline, a total of 480 residents from 28 nursing homes were invited to participate: 286 residents in the intervention group (16 nursing homes) and 184 residents in the comparison group (12 nursing homes). During follow-up, 3 nursing homes withdrew, leaving 25 nursing homes in the final analysis. Among these, 121 residents (43.2%) remained in the intervention group and 65 residents (35.3%) in the comparison group (Fig. 1). From the 9 counties in Lishui, at least 1 nursing home in each county was included in the training program, and the larger nursing homes in each county were considered first for the training program. The comparison group received the intervention later.

The managers at each nursing home identified eligible residents who met the inclusion criteria. To control for selection bias, only one older person in each residence room was selected. Approximately 10–30 older residents per nursing home who were able to answer the questionnaires were included. The inclusion criteria for residents were age ≥ 60 years, living at the nursing home for more than 2 months, and with the ability to fill out a questionnaire independently or with the support of a research assistant.

Sample size estimation followed the recommendation of Polit and Beck [16], with an α of 0.05, power of 0.80, and medium effect size of 0.50. The required sample size was 64 participants per group.

This study included 28 nursing homes in southeast China; only 25 nursing homes were included in the final analysis. Nursing homes provide 24/7 services and care for residents. The residence rooms in the nursing homes are mostly for 2–4 older people, although some also have 1 resident (Table 2). Each residence room contains beds and pieces of furniture. On each floor, cooking facilities and bathrooms are usually shared. In addition, there are common spaces for physical examination and recreation (exercise, billiards, chess, dancing, taiji, drawing, and writing).

### Data collection

Pre-intervention data were collected from July to August 2021 (before the training program), and post-intervention data were collected from October 2023 to January 2024. Data from residents who required assistance were collected by trained postgraduate nursing students who were blinded to the control and intervention groups.

### Intervention

The training program (Chictr.org.cn-ChiCTR2100048628) consisted: knowledge about healthy aging models, integrated medical and older people care service, knowledge, and skills in quality of care, PCC, geriatric symptom assessment, dementia care, environmental enhancement, teamwork skills, staff empowerment, organizational culture, and stress management techniques. The program was developed based on the 13th Five-Year Plan for the Development of National Older People Care Services and the Construction of the Pension System [17], Implementation Methods of Training for Senior Service Personnel [18], and systematic reviews of PCC [6, 7]. The aims of the training program were (1) to improve competency in leading and managing nursing homes among managers, (2) to improve competence among both the manager and staff member in older people care service and PCC, (3) to improve staff member job-career satisfaction, and (4) to improve resident quality-of-life.

The training program was a 24-month educational program for nursing home staff (managers and frontline staff) conducted on-site and online from August 2021 to June 2023. On-site education was conducted over 240 h for 2 years, including 6 stages (Table 1), and 40 h on-site within 1 week. The learning methods employed were lectures, seminars (e.g., case studies, policy or other topic discussions, and sharing experiences, feedback on the baseline scores), nursing skills practice, and field studies. During on-site classes, all of the training content was video recorded and later submitted as online classes in a virtual college system, which was open. This was always available for use by the trainees. In addition to the recorded lectures, supplementary materials—including training manuals, instructional videos, case studies, and dementia care guidelines—were provided online. Trainees also completed assignments such as reflective reports, case-based exercises, and participated in moderated discussion forums to reinforce learning and practical application. On-site lectures were delivered by nurses, psychologists, experts in nursing management, and senior service. Two of the researchers (LX and ZZ) were facilitators of the training program, who answered questions during the on-site classes and supported the trainees in the virtual classes.

**Table 1:** Training program contents for managers and frontline staff in nursing homes

**Stages**	**Contents**		**Training methods**	**Period (On-site class)**
	**Managers**	**Frontline staff**		
Stage 1	1. Professional career planning 2. Healthy aging models 3. Integrated medical and older people care service 4. Policies about nursing homes, such as insurance for long-term care of older people		On-site: lectures and seminars. Online: watching videos, materials, and submitting assignments.Field study: Visiting nursing homes with high scores in the baseline data.	August to September 2021(5 days in July 2021)
Stage 2	1. Marketing, post and salary administration, and E-health management 2. Quality of care, including topics such as **safety**, **PCC,** conflict management. 3. **Researcher feedback about PCC situation**, **quality-of-life of nursing home resident**s (baseline data)**Psyc **	1. First aid and **accidents prevention**, such as falls when walking and from beds, and being lost in the environment 2. Profession risk management, and **PCC skills, such as ADL focused on resident comfort and preferences.** 3. **Researcher feedback about PCC situation**, **quality-of-life of nursing home residents** (baseline data)	On-site: lectures and seminars. Online: watching videos, materials, and submitting assignments.Field study: Visiting nursing homes with high scores in the baseline data.	October to December 2021(5 days in October 2021)
Stage 3	1. Geriatric symptom assessment 2. Nursing care for those with e.g., impaired language and limb function 3. **Dementia care**, such as assessment of cognitive function and pain in persons with dementia, **skill training in specific methods to interact with residents with dementia, verbal and non-verbal communication strategies and multisensory stimulation, and different non-pharmacological alternatives to be used within dementia care taking the older persons perspective and preferences into account**		On-site: lectures and seminars. Online: watching videos, materials, and submitting assignments.Nursing practice: using training rooms at the university for skill training.	March to June 2022 (5 days in March 2022)
Stage 4	1. **Environmental issues**, such as safety improvement, access to and utility of the outdoor environment, and **more variety of social spaces to improve feelings of familiarity** 2. Risk management, such as infection management in nursing home during a pandemic		On-site: lectures and seminars. Online: watching videos, materials, and submitting assignments.Field study: Visiting nursing home in other cities in China.	July to September 2022(5 days in July 2022)
Stage 5	**1. Teamwork and staff empowerment** **2. Organizational culture** 3. **Communication and relationship strategies focusing on older persons’ perspective and preferences**		On-site: lectures and seminars.Online: watching videos, materials, and submitting assignments.	October to December 2022(5 days in October 2022)
Stage 6	**1. Psychological nursing care for older people, such as sleeping problems, older age depression, behavior problems and nursing measurements based on the older persons’ preferences** 2. **Stress management**, such as supporting staff ability to manage stress and burnout related to work, and relaxation techniques		On-site: lectures and seminars. Online: watching videos, materials, and submitting assignments.	March to June 2023 (5 days in March 2023)

Note. Bold text indicates the PCC strategies. ADL, activities of daily living; PCC, person-centered care

### Instruments

Several instruments were used to collect data from the residents and staff. In the present study, PCC data from resident perspectives are presented. To measure PCC, we used the Person-Centered Climate Questionnaire (PCQ) Patient (for the residents) (P).

PCQ-P was developed by Edvardsson et al. [19] and validated by Yoon et al. among nursing home residents [20]. The 17-item Chinese version of the PCQ-P includes the factors hospitality (8 items), safety (5 items), and everydayness (4 items) [20], which are the same factors and items in the English version [20]. The total score is summarized for all the items. The possible scores range from 17 to 119; a high score indicates a more person-centered climate. The factor scores are the sum of all items within each factor. The Chinese PCQ-P used in nursing homes has shown good validity and reliability [21]. Cronbach’s alpha (α) for the present study total score was 0.96, hospitality 0.91, safety 0.87, and everydayness 0.89.

### Data analyses

Data analyses were conducted using IBM SPSS (version 28). Data from four residents were excluded from data analysis because all items for the PCQs were missing. Visual inspection of the histograms and q-q plots of the PCQ-P data revealed no serious deviation from normal distribution.

In previous studies, age [22, 23], sex [22, 23], education [23], marital status [23], income level [23], number of adult children [23], chronic disease [23], and length of residence [20], residence room type [20, 22] and number of residents per nursing homes [24, 25] were significantly related to PCC. Independent sample t-test and chi-square test were used to examine differences in general information (sex, age, education, marital status, having children, main source of income, multimorbidity, length of residence, residence room type, and number of residents/nursing home) and the PCQ-P at baseline between the intervention and comparison groups and between dropouts and participants. PCQ-P levels among participants were higher than those among dropouts (*p* = 0.009); other variables comparing participants and dropouts were not significant. Statistically significant variables at baseline comparing the intervention and comparison groups were treated as covariates in the univariate analysis (ANCOVA) for the effect of the training program (Table 2 and Supplementary 1); Changes in PCQ-P scores between the intervention and comparison groups were compared. To assess the differences in PCQ-P scores over time within the groups, an independent sample t-test was used. To control for potential clustering effects among residents within the same nursing homes, a generalized estimating equation (GEE) was used, with an independent working correlation matrix that has the lowest value when testing the models, also including the above covariates.

**Table 2:** Resident sociodemographic characteristics for the intervention and comparison groups (*N* = 186)

Variables	Total	Groups		*p*-values
		Intervention (*n* = 121)	Comparison (*n* = 65)	
Age (years), Mean (SD), Min-Max	82.2 (8.6) 56–100	82.9 (8.7)	81.0 (8.3)	0.160^a^
Length of residence (years), Mean (SD), Min-Max	3.5 (3.9) 0.1–18	2.7 (2.70)	5.1 (5.1)	**0.003**^**b**^
Number of residents, Mean (SD), Min-Max	140.0 (98.0) 20–328	172.7 (102.1)	79.3 (49.6)	**< 0.001**^**b**^
Sex, n (%)				**< 0.001**^c^
Women	97 (52.2%)	74 (61.2%)	23 (35.4%)	
Men	89 (47.8%)	47 (38.8%)	42 (64.6%)	
Marital status, n (%)				**0.004**^c^
Divorced/single/widow (er)	163 (87.6%)	100 (82.6%)	63 (96.9%)	
Married or in a couple	23 (12.4%)	21 (17.4%)	2 (3.1%)	
Education, n (%)				**< 0.001**^d^
No formal/primary school	132 (71%)	74 (61.2%)	58 (89.2%)	
Junior high school	37 (19.9%)	32(26.4%)	5 (7.7%)	
High school	10 (5.4%)	10 (8.3%)	0 (0.0%)	
College or higher	7 (3.8%)	5 (4.1%)	2 (3.1%)	
Having children, n (%)				**< 0.001**^c^
No	49 (26.3%)	16 (13.2%)	33 (50.8%)	
Yes	137 (73.7%)	105 (86.8%)	32 (49.2%)	
Main source of income, n (%)				**0.026**^c^
Family members or others	116 (62.4%)	68 (56.2%)	48 (73.8%)	
Retirement pension	70 (37.6%)	53 (43.8%)	17 (26.2%)	
Multimorbidity, n (%)				1.000^c^
No	98 (52.7%)	64 (52.9%)	34 (52.3%)	
Yes	88 (47.3%)	57 (47.1%)	31 (47.7%)	
Residence room type (number of residents sharing the same bedroom), n (%)				**0.002**^c^
Double or more	114 (61.3%)	64 (52.9%)	50 (76.9%)	
Single room or a room for couples	72 (38.7%)	57 (47.1%)	15 (23.1%)	

Bold values indicate significant values which were included as covariates to control for potential confounding effects

*SD* standard deviation

^a^Independent t-test, ^b^Mann–Whitney U test, ^c^Chi^2^ test; ^d^Kruskal-Wallis Test

### Ethical considerations

This study was approved by the Medical Ethics Committee of Lishui University (No. 2021-0001). The study details were explained to the participants before obtaining informed consent. Participants received verbal and written study information, and signed an informed consent form. For those not able to write, the researcher signed the informed consent form after obtaining verbal informed consent. Code numbers were used for identification purposes during follow-up. Participants were assured that any data collected would be kept confidential and used only for the present study and informed that they could drop out.

## Results

### Sociodemographic characteristics

The sociodemographic characteristics of residents, including comparisons between the two groups, are presented in Table 2. The sociodemographic data for residents in the intervention and comparison groups differed significantly in terms of sex, marital status, education, having adult children, income sources, length of residence, room type, and resident/nursing home distribution. Residents in the intervention group had a higher number of women, higher levels of education, were more likely to have adult children, and more often received retirement pensions than residents in the comparison group. In the overall sample, the majority were divorced/single/widowed d and the mean age was 82.2 (SD = 8.6) years. Most lived in rooms with 2 or more persons and had lived there for more than 3.5 years (SD = 3.9) (Table 2).

### Person-centered care

Changes in PCQ-P scores over time showed significant differences between groups. The intervention group improved over time (mean change + 13.6, SD = 20.0), with scores increasing from 91.1 (SD = 16.2) at baseline to 104.7 (SD = 12.9) post-intervention. In contrast, the scores of the comparison group declined (mean change − 5.9, SD = 24.6), with scores decreasing from 97.4 (SD = 15.3) to 91.5 (SD = 17.3). These differences were significant in both unadjusted and adjusted analyses (*p* < 0.001 for both) (Table 3). Significant between-group differences were found for all three factors: hospitality, safety, and everydayness (adjusted ANCOVA *p* < 0.001 for all; Table 3). The intervention group showed improvement among all the factors over time (*p* < 0.001). Whereas the comparison group declined in everydayness (*p* = 0.018). Using the GEE models, the differences in changes over time between the two groups remained significant for the total score and the three factors of the PCQ-P (all *p*-values for GEE < 0.001) (Table 3).

**Table 3:** Comparison of Person-centered climate Questionnaire-Patient version score changes (pre- and post-test) between the intervention and comparison groups (the interaction effect) (*N* = 186)

Variables	Intervention group			Comparison group			(*p*-values)^a^	(*p*-values)^b^	(*p*-values)^c^
	Pre-test Mean (SD)	Post-test Mean (SD)	*p*-values for the within effect	Pre-test Mean (SD)	Post-test Mean (SD)	*p*-values for the within effect			
Person-centered climate	91.1 (16.2)	104.7(12.9)	**< 0.001**	97.4 (15.3)	91.5 (17.3)	0.059	**< 0.001**	**< 0.001**	**< 0.001**
Hospitality	42.0(7.9)	49.2 (6.1)	**< 0.001**	45.4 (7.2)	43.1 (8.2)	0.119	**< 0.001**	**< 0.001**	**< 0.001**
Safety	27.4 (4.7)	30.8 (3.9)	**< 0.001**	28.7 (4.9)	27.0 (5.1)	0.080	**< 0.001**	**< 0.001**	**< 0.001**
Everydayness	21.3 (4.5)	24.7 (3.1)	**< 0.001**	23.2 (3.9)	21.3 (4.1)	**0.018**	**< 0.001**	**< 0.001**	**< 0.001**

Bold values indicate significant values

*SD* standard deviation

^a^Independent samples t-test comparing the change scores between the intervention and comparison groups, non-adjusted test

^b^ANCOVA test comparing the change scores for the intervention and comparison groups controlling for sex, education, marital status, income source, number of adult children, length of residence, type of room, and residents/nursing homes

^c^Generalized estimating equation parameter estimates controlling for sex, education, marital status, main source of income, having children, length of residence, type of room, and number of residents/nursing homes

## Discussion

To the best of our knowledge, this was the first study to implement a PCC training program in Chinese nursing homes. The results indicated that the training program increased the total and factor scores of the PCQ-P, which were higher in the intervention group than in the comparison group. The comparison group had lower scores for everydayness as rated by nursing home residents over time.

Our positive results for a person-centered climate from residents in the intervention group are consistent with those of previous studies [15]. Most prior studies have assessed the effectiveness of PCC staff education programs from the residents’ perspective using proxy evaluations by staff [10] and next of kin [8, 9]. Compared with proxy-rated assessments, self-reporting is considered the gold standard. In this context, we highlight studies that specifically examined between-group changes in resident-reported outcomes following educational interventions. For instance, Pakkonen et al., [9] demonstrated residents reported improvements in the total score of the person-centred climate scale, and in the subscales of safety and hospitality. The intervention included a 13-hour education among nurses in long-term care of older people delivered over 10 weeks. The intervention included lectures, brainstorming sessions, and other interactive activities. Similarly, Roos et al., [15] conducted an educational intervention consisting of eight seminars over six months, implemented among staff in residential facilities. The intervention focused on topics such as residents’ dignity, well-being, working methods of the staff, and the development and evaluation of improvement plans. Improvements were observed in the resident-reported total score of the person-centered climate scale and the subscales of safety, everydayness, and hospitality.

The intervention was developed based on national and regional guidelines for older people’s care service; and previous research evidence [6, 7] about PCC, which covered respect for older peoples’ values, preferences, expressed needs, interaction and integration of care, teamwork, staff empowerment, environmental enhancement, and emotional support. Unlike previous PCC intervention studies [8–10], stakeholder engagement (Ministry of Civil Affairs in Lishui and staff from nursing homes) was employed in the development and implementation of the training program in the present study. The engagement of stakeholders, such as government staff and staff members in nursing homes, in the development of an intervention can improve the acceptability of a program and facilitate changes [26, 27]. Support for translating guidelines into practice is another important factor [28]. The inclusion of one manager per training program from each nursing home, together with one staff member, may also have been a reason for our positive outcomes.

The participants received feedback on the PCC scores and quality of life of the nursing home residents (pre-test) in the intervention group. The total score and factor scores of person-centered climate, as rated by residents before the intervention, were lower than other studies [20, 29]. Notably, the dimensions of hospitality and everydayness received the lowest ratings. Specific items such as “staff are knowledgeable”,“it feels like home”,“staff make extra efforts for my comfort” and “I can get that little bit extra” scored particularly low. In response, the staff reflected on potential reasons for these low scores and discussed targeted strategies for improvement, including enhancing the physical environment, respecting resident preferences and needs, and strengthening interpersonal interaction skills.

Regarding within-group changes in everydayness factors, the comparison group showed a decline in scores over time. Everydayness refers to experiences of a de-institutionalized environment containing aspects of improved familiarity, a more home-like atmosphere [20]; and opportunities for residents to keep up with current events and interact other than their illnesses [19]. Following the Plan for the Development of National Older People Care Services [17]; construction of older people-friendly living environments in China was enhanced. Augmentation of the physical environment in nursing homes has mostly focused on the objective function of the environment, prevention of accidents, more paintings, and outdoor flowers. Resident preferences and needs are often neglected or forgotten [30].

### Methodological consideration

The assignment of participants to the intervention and comparison groups was not random; it was based on the distribution across all the counties of the city and the number of beds in those nursing homes. The quasi-experimental design, without random assignment, may have introduced selection bias. For example, the comparison group showed higher baseline scores on the PCQ-P, suggesting that these nursing homes may have a more person-centered care culture before the intervention. This baseline imbalance could have influenced the magnitude of observed intervention effects. To control for this, we compared the groups at baseline, and the variables that differed were controlled for in the analyses of the intervention effect. Future studies using cluster-randomized controlled trials are recommended to strengthen causal inference. The relatively high dropout rate, similar to existing literature, may limit the generalizability of the findings. Residents who completed the study reported higher baseline PCQ-P scores compared to those who dropped out. This pattern suggests that participants with more favorable perceptions of person-centered care may have been more likely to remain engaged. This could have potentially led to an overestimation of the intervention’s effectiveness. In the present study, the reasons for dropping out were mostly death, inability to communicate, and leaving the nursing homes (Fig. 1).

The strengths of this study were that validated instruments were used for PCC, rated by residents, and they had good internal consistency. The data collectors were blinded as to whether the nursing homes were in the intervention group or not. A GEE was used to control for possible clustering effects among residents within the same nursing home, and significant results from the ANCOVA remained.

### Clinical implications

The implementation of national and regional guidelines for older people’s care services improved the person-centered climate as rated by the residents in nursing homes. The positive outcomes indicated that it is essential for stakeholders, such as government staff and nursing home managers, to engage in interventions and invest resources to facilitate changes. It is also important that staff receive feedback about PCC scores and the quality of life of their nursing home residents to improve PCC. Furthermore, staff members must diffuse their knowledge to coworkers at their nursing homes.

## Conclusion

Training programs, including knowledge and skills about PCC, dementia care, teamwork, staff empowerment, and psychological nursing care skills, are associated with improvement in a person-centered climate as rated by residents.

## Supplementary Information

Supplementary Material 1.

## Data Availability

The datasets used and/or analyzed during the current study are available from the corresponding author on reasonable request.

## Abbreviations

PCC: Person-centered care
PCQ-P: Person-Centered Climate Questionnaire Patient
ANCOVA: Univariate analysis
GEE: Generalized estimating equation
