# Supplementary material for: Effects of a training program on person-centered care in nursing homes rated by residents: a quasi-experimental design
Source: BMC Geriatr. 2025 Oct 23;25:799. doi: 10.1186/s12877-025-06340-7 (PMC12548235; doi:10.1186/s12877-025-06340-7)
Supplement: Supplementary file 1 — Supplementary Material 1. [file 12877_2025_6340_MOESM1_ESM.docx]

Supplementary 1. Covariates in ANCOVA and GEE model

| **Variables** |
| --- |
| Length of residence (years) |
| Number of residents |
| Sex (women, men) |
| Marital status (divorced/single/widow (er), married or in a couple) |
| Education (no formal/primary school, junior high school, high school, college or higher |
| Having children (no, yes) |
| Main source of income (family members or others, retirement pension0 |
| Residence room type (double or more, single room or a room for couples) |
